# TSSNote-CyaPromBERT: Development of an integrated platform for highly accurate promoter prediction and visualization of *Synechococcus* sp. and *Synechocystis* sp. through a state-of-the-art natural language processing model BERT

**DOI:** 10.3389/fgene.2022.1067562

**Published:** 2022-11-29

**Authors:** Dung Hoang Anh Mai, Linh Thanh Nguyen, Eun Yeol Lee

**Affiliations:** Department of Chemical Engineering (BK21 FOUR Integrated Engineering Program), Kyung Hee University, Yongin-si, South Korea

**Keywords:** deep learning, natural language processing, transformer, promoter prediction, dRNA-Seq, differential RNA sequencing

## Abstract

Since the introduction of the first transformer model with a unique self-attention mechanism, natural language processing (NLP) models have attained state-of-the-art (SOTA) performance on various tasks. As DNA is the blueprint of life, it can be viewed as an unusual language, with its characteristic lexicon and grammar. Therefore, NLP models may provide insights into the meaning of the sequential structure of DNA. In the current study, we employed and compared the performance of popular SOTA NLP models (i.e., XLNET, BERT, and a variant DNABERT trained on the human genome) to predict and analyze the promoters in freshwater cyanobacterium *Synechocystis* sp. PCC 6803 and the fastest growing cyanobacterium *Synechococcus elongatus* sp. UTEX 2973. These freshwater cyanobacteria are promising hosts for phototrophically producing value-added compounds from CO_2_. Through a custom pipeline, promoters and non-promoters from *Synechococcus elongatus* sp. UTEX 2973 were used to train the model. The trained model achieved an AUROC score of 0.97 and F1 score of 0.92. During cross-validation with promoters from *Synechocystis* sp. PCC 6803, the model achieved an AUROC score of 0.96 and F1 score of 0.91. To increase accessibility, we developed an integrated platform (TSSNote-CyaPromBERT) to facilitate large dataset extraction, model training, and promoter prediction from public dRNA-seq datasets. Furthermore, various visualization tools have been incorporated to address the “black box” issue of deep learning and feature analysis. The learning transfer ability of large language models may help identify and analyze promoter regions for newly isolated strains with similar lineages.

## Introduction

A classic problem in bioinformatics is the challenge of predicting promoters (Zhang et al., 2022). Promoter regions are DNA regions where RNA polymerase binds to initiate the transcription process, the first step in the central dogma of molecular biology (Butler and Kadonaga, 2002). Owing to their essential role in regulating and determining the timing and expression levels of genes needed for vital functions, the prediction and in-depth functional analysis of promoters have been of interest to biologists. Previously, owing to the complexity of cis-regulation networks and lack of data, attempts at developing promoter prediction tools were inadequate (Bhandari et al., 2021). However, recent advancements in machine learning and deep learning have successfully leveraged genomic data. To date, many groups have successfully constructed promoter prediction tools using traditional machine learning methods, knowledge-based position matrix weight (Huerta and Collado-Vides, 2003; Burden et al., 2005; Rangannan and Bansal, 2010; Di Salvo et al., 2018) through support vector machines, and artificial neural networks for this logistic regression task (Gordon et al., 2003; da Silva et al., 2006; Mann et al., 2007; Towsey et al., 2008; He et al., 2018; Liu et al., 2018; Rahman et al., 2019; Xiao et al., 2019; Zhang et al., 2019; Li et al., 2021). Convolutional neural networks (CNN) and recurrent neural network (RNN)-based architectures (long short-term memory, gated recurrent units) have recently become the most popular choices for promoter classification (Nguyen et al., 2016; Le et al., 2019; Oubounyt et al., 2019; Amin et al., 2020; Zhu et al., 2021). CNN-based models depend on predetermined kernel size designs to extract and generalize local features; therefore, they might fail to capture long-range contexts. To overcome this limitation, some research groups have integrated RNN-based models to retrieve long-term dependencies. By design, LTSM computations from RNNs are processed sequentially and depend on the outputs of the previous hidden states for the next state to maintain the sentence structure and context; however, this, in turn, leads to the vanishing gradient problem. These limitations pose difficulties and may restrict the scalability and flexibility of constructed models when applied to other species.

Since its first appearance in 2017, the transformer architecture, with its unique self-attention mechanism, has revolutionized the natural language processing (NLP) field and achieved SOTA performance in various machine learning tasks (Vaswani et al., 2017). As these transformers perform well, they have made their way to other branches (e.g., computer vision) (Wu et al., 2020; Arnab et al., 2021; Zhou et al., 2021) that were previously dominated by CNNs, and they are now also used in multimodal learning for content generation (Tsai et al., 2019; Yu et al., 2019; Dzabraev et al., 2021). Transformer-based models are versatile and can be incorporated into different architectures owing to their robustness and flexibility through their learning-transfer capability. Considering the sequential nature of DNA, which can be regarded as a natural language with unique grammar and lexicon, transformer-based models are particularly well suited for supervised classification tasks.

Therefore, adopting a different approach in the current study, we employed and compared transformer-based models for the promoter prediction problem. To date, most of the currently constructed models have been designed for popular species with curated regulatory databases such as humans, fruit flies, mice, *Escherichia coli*, and yeasts (Oubounyt et al., 2019; Rahman M et al., 2019; Li et al., 2021). However, there is still considerable interest in integrating deep-learning techniques for promoter analysis in other (less popular) species. For example, cyanobacteria are an ancient and diverse group of photo-oxygenic prokaryotes with ample potential for the photosynthetic production of value-added chemical compounds from the greenhouse gas CO_2_. Many cyanobacterial species with a high potential for valorizing CO_2_ are still being isolated and characterized every year. Some of the most notable genera were *Synechocystis* and *Synechococcus*. These model organisms can convert CO_2_ into various useful products (Luan et al., 2019; Sarnaik et al., 2019; Lin et al., 2020; Pattharaprachayakul et al., 2020; Qiao et al., 2020; Taylor and Heap, 2020; Kato and Hasunuma, 2021; Roh et al., 2021; Santos-Merino et al., 2021). Although they have been characterized and researched for a few decades, the application of deep learning for promoter prediction specifically in cyanobacteria is still lacking. Therefore, in this study, we used the promoters of *Synechococcus elongatus* sp. UTEX 2973, the fastest growing cyanobacterium for model training and testing (Song et al., 2016; Mueller et al., 2017). We further conducted cross-validation of the promoters of the model organism *Synechocystis* sp. PCC 6803 to test whether the models also work on related species (Ikeuchi and Tabata 2001). Combined with knowledge-based analysis, in-depth model characterization may help tackle the “black box” problem of deep-learning models.

To facilitate the development and incorporation of SOTA transformer-based promoter prediction tools, we reconstructed a pipeline (using TSSNote and PromBERT Google Colab notebooks) to compute and extract the promoters of public differential RNA-seq (dRNA-seq) datasets from the National Center for Biotechnology Information Sequence Read Archive (NCBI SRA) database and used them for model training. dRNA-seq is an RNA sequencing technique that allows the determination of TSS at 1 bp resolution by enriching primary transcripts (Bischler et al., 2015). In contrast to conventional differential expression RNA-seq (RNA-seq), dRNA-seq requires additional treatments and more expensive and complex procedures, making these datasets rather limited. Transfer learning is a core advantage of large-parameter language models. We expect that, with fine-tuning, transformer-based promoter models can be good approximators for other related species. To improve the accessibility to researchers with and without expertise in machine learning, separate modules of the pipeline for promoter extraction, model training, promoter prediction, and visualization were ported into the cloud-based platform Google Colab. We demonstrated that, even without the advantage of the pre-training phase, transformer-based models, such as bidirectional encoder representations from transformers (BERT) and XLNET, are capable of highly accurate promoter prediction for *Synechocystis* and *Synechococcus* species solely through a context-wise self-attention mechanism (Devlin et al., 2018; Yang et al., 2019).

## Materials and methods

### Datasets

Raw dRNA-seq datasets for *Synechocystis* sp. PCC 6803 and *Synechococcus elongatus* sp. UTEX 2973 and for *Synechocystis* sp. PCC 6714 were downloaded from the NCBI SRA database, and genomic DNA sequence assemblies were downloaded from the NCBI RefSeq database (Table 1).

**TABLE 1:** Datasets employed in this study.

Species	SRA accession number	Condition	TEX treatment
*Synechococcus elongatus* sp. UTEX2973	SRR6334749, SRR6334750	Primary transcripts under normal condition	TEX (+)
	SRR6334747, SRR6334748	Control under normal condition	TEX (-)
*Synechocystis* sp. PCC 6803	SRR1019366, SRR1019365	Primary transcripts under exponential and stationary phase	TEX (+)
	SRR1019368, SRR1019367	Secondary reads from 10 different conditions	TEX (-)
*Synechocystis* sp. PCC 6714	SRR1019241	Primary reads from stationary phase	TEX (+)
	SRR1019242	Secondary reads from 10 different conditions	TEX (-)

Independent *E. coli* promoter datasets for benchmarking were obtained from https://github.com/chenli-bioinfo/promoter.

Available data and local and Google Colab versions of TSSNote-CyaPromBERT are available at https://github.com/hanepira/TSSnote-CyaPromBert.

### Constructing promoter extracting module from dRNA-seq datasets

**FIGURE 1:**
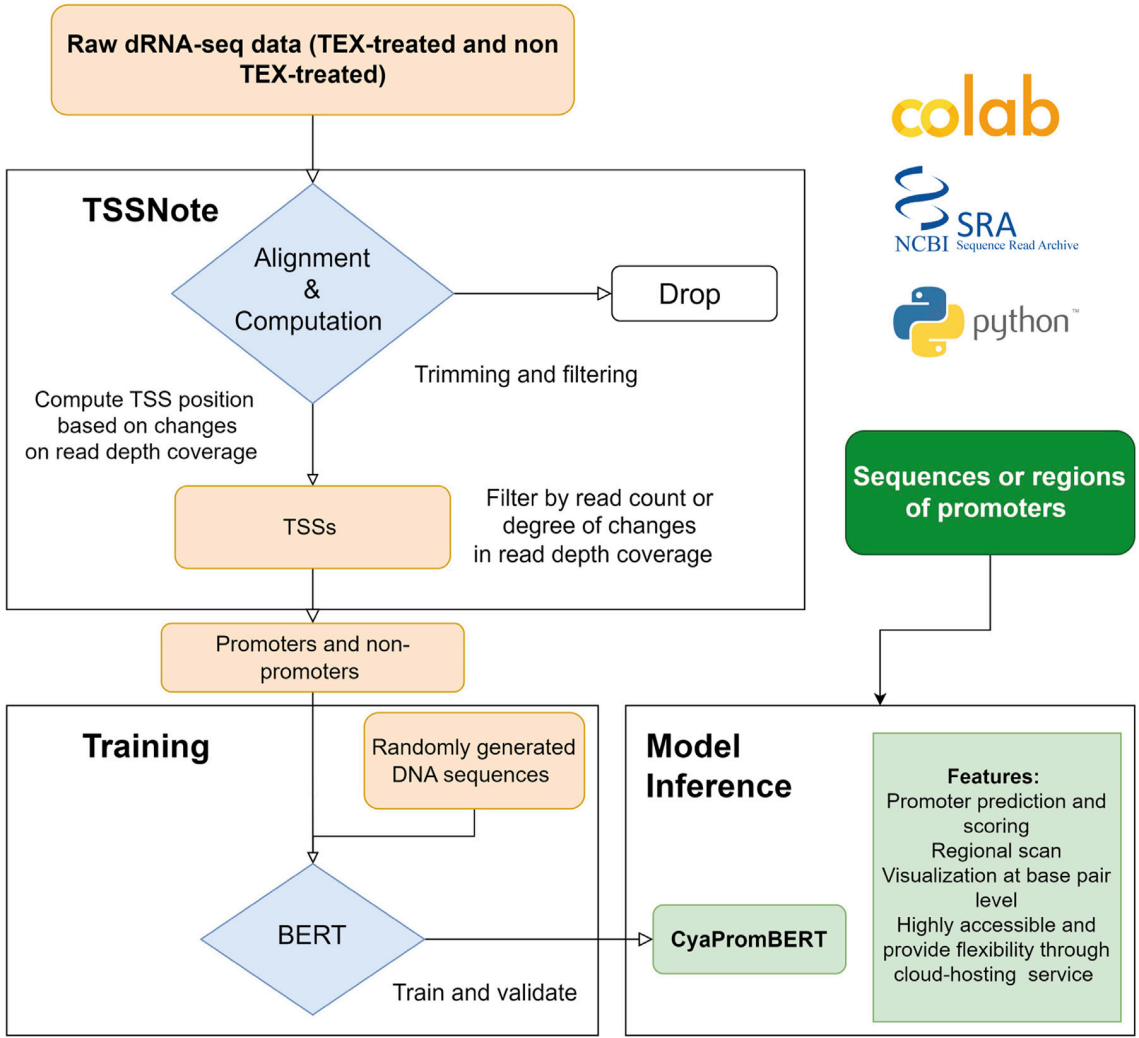
Overall scheme for constructing and developing TSSNote and CyaPromBERT. TSSNote facilitates downloading raw dRNA-seq datasets from NCBI SRA database and conducts alignment, sorting, and filtering for extracting promoters and non-promoters. These sequences are later used to train a BERT model for the task of promoter prediction. Randomly generated DNA sequences with similar size to promoter length are added to reduce biases, and overfitting is used to improve the model’s robustness. The trained model is capable of promoter prediction, regional scanning, and visualization at base-pair level.

**FIGURE 2:**
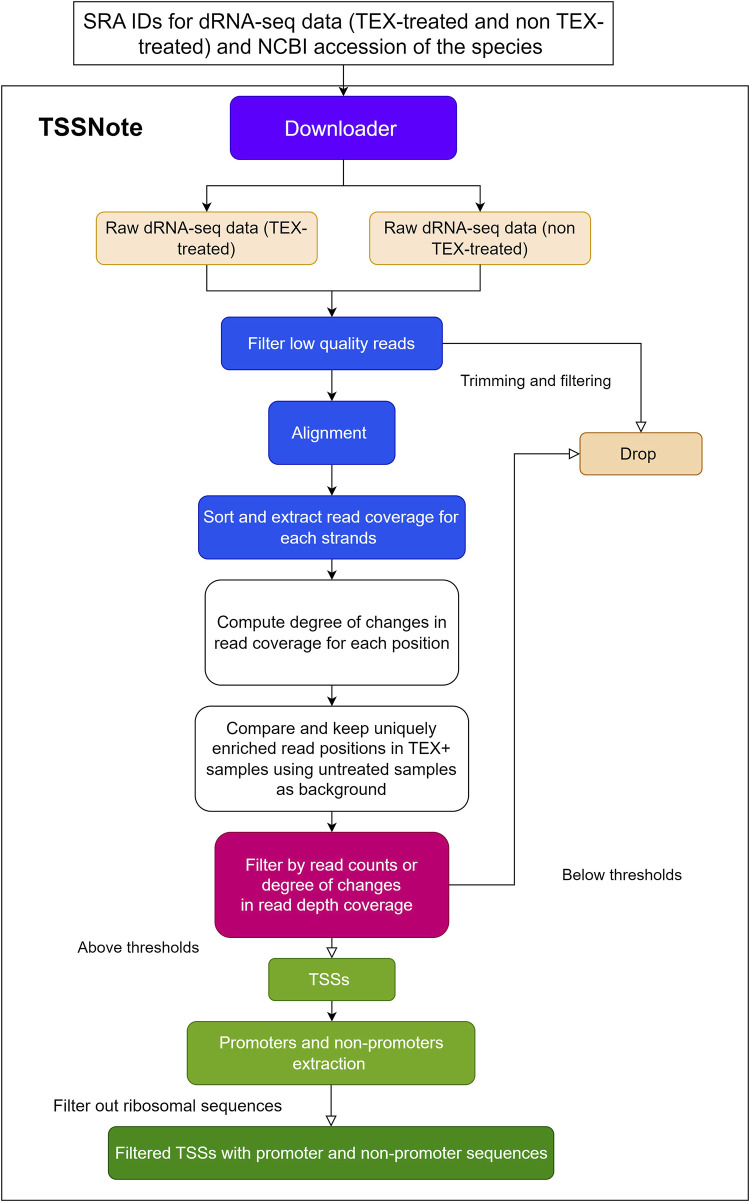
Detailed flowchart of TSSNote operation to extract TSSs, promoters, and non-promoter sequences.

Because one of the objectives of the current work is to create a cloud-computing-based pipeline that can be applied without strong hardware requirements, we implemented algorithms in a Colab notebook for TSS prediction based on changes in read coverage, in a similar manner to TSSpredator (Dugar et al., 2013) but with more flexibility for customizations. This promoter extracting module (TSSNote) takes SRA ids for TEX (+) and TEX (-) treatments and fasta from NCBI as inputs and conducts alignment by HISAT2 and read coverage extraction through SAMTools. HISAT2 enables soft-clipping alignment, through which adapters do not interfere with the read alignment. SAMTools are then used to extract read coverage from the plus and minus strands for later computations. The read coverage files from both TEX (+) enrichment and TEX (-) were used to locate and compute the potential TSSs enriched by TEX treatment. Because the quality of dRNAseq datasets is dependent on experimental procedures, after calculating potential TSSs, users can filter TSSs based on the read coverage cut-off or coverage change cut-off. BAM files can be downloaded into local drives for manual observation and curation using NGS genome browsers. The overall design is illustrated in Figure 1, and the detailed workflow of the TSSNote is shown in Figure 2.

Read coverage change at a specific location is calculated by the following function:

$$△xi=\frac{x_{i+1}+c}{x_{i}+c}$$

Where: $x_{i}$ = coverage depth at position i $x_{i}+1$ = coverage depth at position i+1 $∆x_{i}$ = change factor from xi to $x_{i}+1$ c = calibration constant to prevent division zero (0.01).

### Promoter and non-promoter sequences extraction

Promoters were extracted directly upstream from the predicted TSSs. For promoter sequences, ribosomal RNA depletion in dRNAseq experiments may not be 100%; therefore, further trimming methods were implemented. We tested the TSSs identified by TSSNote based on the wildtype dataset with the TSSs proposed in the original publication (Tan et al., 2018). Even though the implementation method was different, many of the predicted TSSs were consistent. By setting constraints more stringent, through expression strength and degree of changes, more than 90% of the TSSs identified in the wildtype dataset were also found in the original proposed TSSs concatenated from multiple conditions. Therefore, filtered promoter datasets extracted from strongly expressed and enriched TSSs should be sufficiently reliable. As deep-learning models require a large amount of data for accurate generalization, we believe that the flexibility offered by TSSNote can be crucial. Furthermore, read counts and fold-changes in read coverage can provide more information to group and filter promoters based on promoter strength. It can be used independently or together with existing tools for better analysis. In the current work we lowered the constraints to take into account the potential spurious transcriptional events and weak promoters of other sigma factor groups which would be filtered by the method used in the original publication. The good performance on cross validation and clear pattern enrichment indicate that the model has successfully learned key features from the extracted promoters for promoter recognition task.

The non-promoter sequences were extracted from the “non-promoter” regions. Specifically, Non-promoter sequences were sampled from the downstream of TSSs. If the distance between two neighboring TSSs is larger than 2 times the sequence length, that interval region is marked and used for sampling non promoter sequences. We further added 10% randomly generated sequences to increase noise and reduce overfitting. The non-promoter sequences then are shuffled, and a portion of the non-promoter sequences was used at the ratio 1:1 promoter–non-promoter for model training.

### Model training

The TSSs of each species from different datasets was extracted and concatenated for model construction using Python wrapper TSSNote, which was written in Python 3.9 as a user-friendly pipeline to conduct raw data gathering using SRA toolkits 3.0 (https://trace.ncbi.nlm.nih.gov/Traces/sra/sra.cgi?view=software) and Entrez-direct (Kans 2022), sequence indexing, read alignment by HISAT2 (Kim et al., 2019), strand sorting, and read coverage calculation by SAMtools (Danecek et al., 2021). Promoter sequences were extracted from the calculated TSSs using the Biopython package (Cock et al., 2009).

To construct CyaPromBert and evaluate the performance of different transformer-based models, Pytorch 1.11.0 and Pytorch-lightning 1.6.4 (Paszke et al., 2019). Transformer-based models were constructed using base models from huggingface’s transformer library 4.18.0 (Wolf et al., 2020).

The probability was calculated by the sigmoid function:

$$S\left(x\right)=\frac{1}{1+e-x}$$

The performance of the models was evaluated by precision, recall, F-1, and AUPRC, AUROC scores.

$$Precision=\frac{tp}{tp+fp}$$

$$Recall=\frac{tp}{tp+fn}$$

$$F1=\frac{2tp}{2tp+fp+fn}$$

Where: tp = true positive fp = false positive fn = false negative

The area under the precision-recall curve (AUPRC) is calculated from the average precision score and AUROC is the area under the receiver operating characteristics.

Binary cross entropy was used as the loss function.

$$BCELoss=-\frac{1}{N}\overset{N}{\underset{i=1}{\sum }}\left(yi*\log(pi\right)+\left(1-yi\right)*\log\left(1-pi\right))$$

Attention weight visualization libraries, BERTviz 1.4.0, and Captum 0.5.0, were implemented to improve visualization and interpretability (Vig 2019; Kokhlikyan et al., 2020). Both TSSNote and the models were first developed and trained on a local workstation equipped with an NVIDIA RTX 3070 before porting and testing on the Google Colab cloud computing service.

## Results and discussion

### Selecting the best performing SOTA transformer-based model for promoter prediction

**FIGURE 3:**
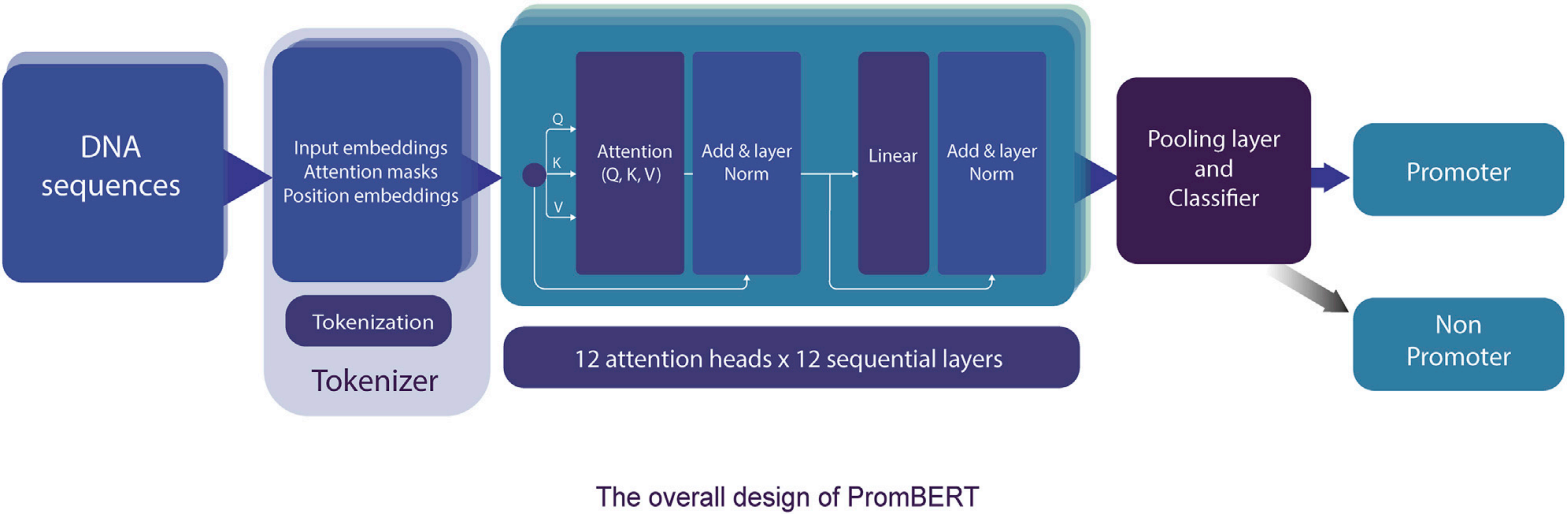
The detailed model architecture of PromBERT for promoter prediction. DNA sequences of fixed length are tokenized using a custom 1bp tokenizer and fed into 144 attention modules. Based on the final tensors in the pooling layer, the classifier calculates the probabilities of promoter and non-promoter using the sigmoid function. Backpropagation was conducted using binary cross entropy loss.

**FIGURE 4:**
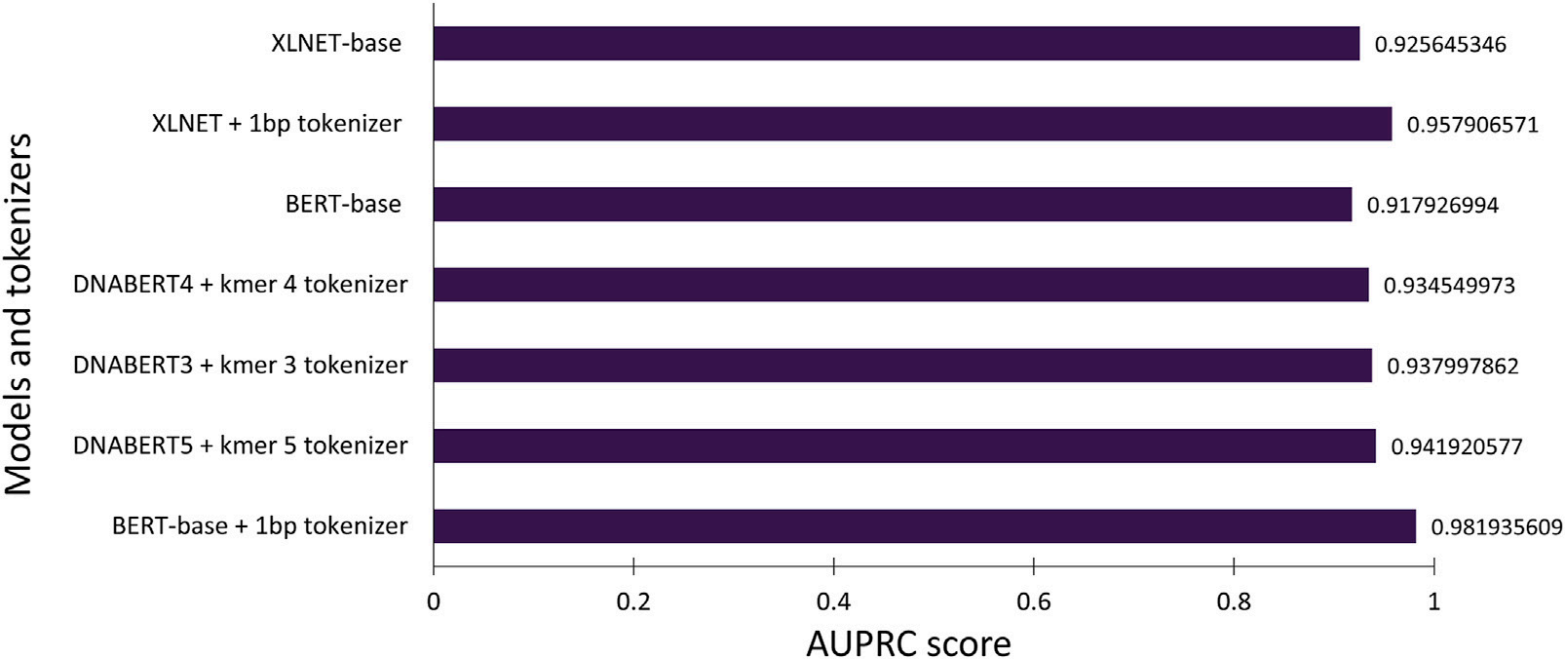
Average precision scores of the tested transformer-based models.

The transformer-based architecture has demonstrated that, with sufficient data, matrix multiplications, linear layers, and layer normalization, the deep-learning model can achieve SOTA machine translation tasks without relying on CNN and RNN (Vaswani et al., 2017). BERT and XLNET are two of the most popular transformer-based language models (Devlin et al., 2018; Yang et al., 2019). Fundamentally, these large-language models are stacks of encoding modules from the original transformer model. However, they are pre-trained differently and use different tokenizers. BERT is an autoencoding-based model, whereas XLNet employs an autoregressive method similar to the famous GPT models from OpenAI (Floridi and Chiriatti 2020). These differences reflect the capability to capture the semantic context for prediction in masked language prediction pretraining, and thus they can affect the performance of the model. However, the corpora, on which both BERT and XLNet were trained, are far different from the genomic DNA sequences; therefore, they might not have pretraining advantages. Thus, we also compared a different variant of BERT (DNABERT) pretrained on human genomic DNA at different kmer lengths (from three to five nucleotides) (Ji et al., 2021). The DNABERT models outperformed previous CNN-based models for TATA and non-TATA promoter prediction tasks in eukaryotes. To improve the resolution, we trained a byte-level byte-pair-encoding (BPE) tokenizer at a length of one nucleotide (or kmer 1). The operating mechanism is illustrated in Figure 3 and the performance results are listed in Table 2 and Figure 4.

**TABLE 2:** Performance of popular transformer-based NLP models for promoter prediction.

Model and tokenizer	AUROC		Precision		F1 score		Support	
	Promoter	Non-promoter	Promoter	Non-promoter	Promoter	Non-promoter	Promoter	Non-promoter
XLNET	0.926	0.925	0.85	0.84	0.85	0.85	1018	1019
XLNET + 1bp tokenizer	0.97	0.97	0.92	0.92	0.92	0.92	1018	1019
BERT-base	0.941	0.942	0.84	0.89	0.87	0.87	1001	1036
BERT-base + 1bp tokenizer	0.977	0.977	0.92	0.95	0.93	0.93	1001	1036
DNABERT3 + kmer 3	0.944	0.944	0.9	0.84	0.86	0.88	1008	1029
DNABERT4 + kmer 4	0.944	0.944	0.88	0.86	0.87	0.87	1028	1009
DNABERT5+ kmer 5	0.956	0.956	0.9	0.89	0.89	0.89	1031	1006

For this particular promoter prediction task (using binary cross entropy as the loss function and F1 score as the key determinants to evaluate model performance), both XLNet-base and BERT-base using a one kmer length byte-level BPE tokenizer had the best performance compared to the default tokenizers or tokenizer at different lengths. Both XLNet+1bp tokenizer and BERT+1bp tokenizer achieved AUROC scores of 0.97 and 0.977, and F1 scores of 0.92 and 0.93 respectively. These two models exhibited comparable performance. However, during training and testing, XLNet used more computing resources than BERT; therefore, we selected the BERT-base + 1bp tokenizer for further investigation. The corpora in which these two base models were pretrained did not contain genomic databases. They should not benefit from the pre-training process for the promoter prediction task. The high performance can be attributed to context awareness (context-based embedding) of the position and composition of the tokens (nucleotides) through the self-attention mechanism. We further tested the performance of the BERT-base + 1bp tokenizer and DNABERT5 + 1bp tokenizer. The results further show that there are no differences in performance. These findings also confirmed that, during training for promoter prediction tasks using BERT, the choice of tokenizer influenced the performance.

Surprisingly, the DNABERT variants trained in the genomic context performed worse than the BERT-base + 1bp tokenizer. Longer kmer lengths might provide a better context and have more meaningful biological values for interpretation (Ji et al., 2021); however, the F1 scores of the pretrained DNABERT 3, 4, and five were lower than those of BERT-base and XLNet with the 1bp tokenizer. One possible explanation for this finding is that the 1bp tokenizer better captured nuances at the single-nucleotide level interactions in the training dataset. As the promoter datasets in the current study were extracted solely from TSSs and were not grouped in transcriptional factor classes, less information is required to make decisions. This model may significantly favor specific nucleotides at certain fixed positions. Using tokenizers with longer kmer lengths (for the case of DNABERT) might be better for other genomic applications or designs that require larger curated datasets with expected long-range interactions within those genomic sequences. This is particularly relevant if the model is pre-trained or fine-tuned by permutation and masked language modeling first on the genomic data of the target species. We further tested the influence of promoter length on model performance; however, increasing the promoter length to 200bp did not change the performance of any of the tested models (data not shown).

### Evaluating model performance compared to existing promoter prediction models using independent datasets from *E. coli*

To evaluate the robustness of the proposed BERT-base +1bp tokenizer for promoter prediction task, we conducted model training using an independent dataset for σ70 promoters for model benchmarking from a previous study (Zhang et al., 2022).

**FIGURE 5:**
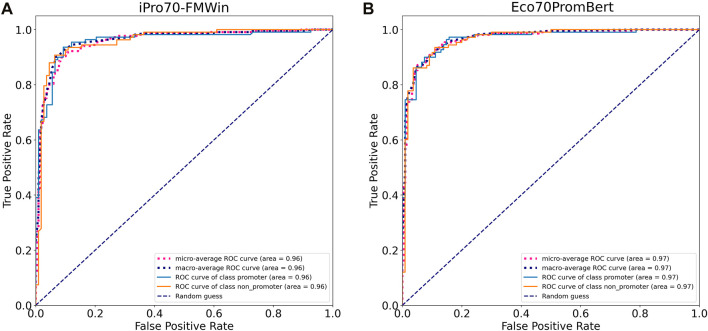
Model performance based on receiver operating characteristic curves tested on an independent promoter datasets from *E. coli*. **(A)** iPro70-FMWin **(B)** Eco70PromBERT- (BERT-base + 1bp tokenizer trained on promoter datasets from *E. coli*).

We compared the performance of our model with two promoter prediction webservers iPro70-FMWin (Rahman et al., 2019) and iPromoter-2L2.0 (Liu et al., 2018) which were reported to have very high accuracy for σ70 promoters. The results showed that those three models performed equally well on the benchmarking dataset with F1 scores around 91%. Our model performed slightly better across promoter and non-promoter tag (Table 3). Since iPro70-FMWin also provides probability scores, we compared the AUPRC scores of this model with our Eco70PromBERT-1bp (Figure 5). Our model had a better AUPRC score of 0.967 compared to 0.953 from iPro70-FMWin.

**TABLE 3:** Performance of Eco70PromBERT and popular promoter prediction models for *E.coli* using an independent dataset (σ70 promoters and non-promoters).

Model and tokenizer	AUROC		Precision		F1 score		Support	
	Promoter	Non-promoter	Promoter	Non-promoter	Promoter	Non-promoter	Promoter	Non-promoter
Eco70PromBERT (BERT-base + 1bp tokenizer)	0.92	0.90	0.91	0.91	0.91	0.91	110	108
iPro70-FMWin	0.90	0.90	0.93	0.88	0.90	0.91	110	108
iPromoter-2L2.0	0.91	0.91	0.90	0.92	0.91	0.91	110	108

The results illustrated the robustness of BERT-base + 1bp tokenizer for promoter prediction task in general. Considering that both iPro70-FMWin and iPromoter-2L2.0 were designed specifically to extract sequence features with various customizations for promoter classification to achieve SOTA performance. The plug-and-play characteristic of large language models like BERT would be better for scalability and broader applications.

### Interpreting the model’s behavior through Monte Carlo sampling and attention score visualization

Interpreting deep-learning (DL) models is another important aspect of model validation. One of the main issues concerning deep-learning models is the “black box” problem, where users might not know how DL models process and compute the outputs for reverse engineering and understanding. This problem is particularly difficult for large parameter models such as NLP models (e.g., BERT). Specifically, the BERT-base model used in this study consists of 86.8 million trainable parameters from 144 attention modules (12 layers × 12 heads). The use of attention scores to visualize token weights is a commonly used method for improving model understanding. We employed integrated libraries for interpretability, namely BERTviz and Captum, to gain more insight into CyaPromBERT behavior and key features determining true promoters or non-promoters.

**FIGURE 6:**
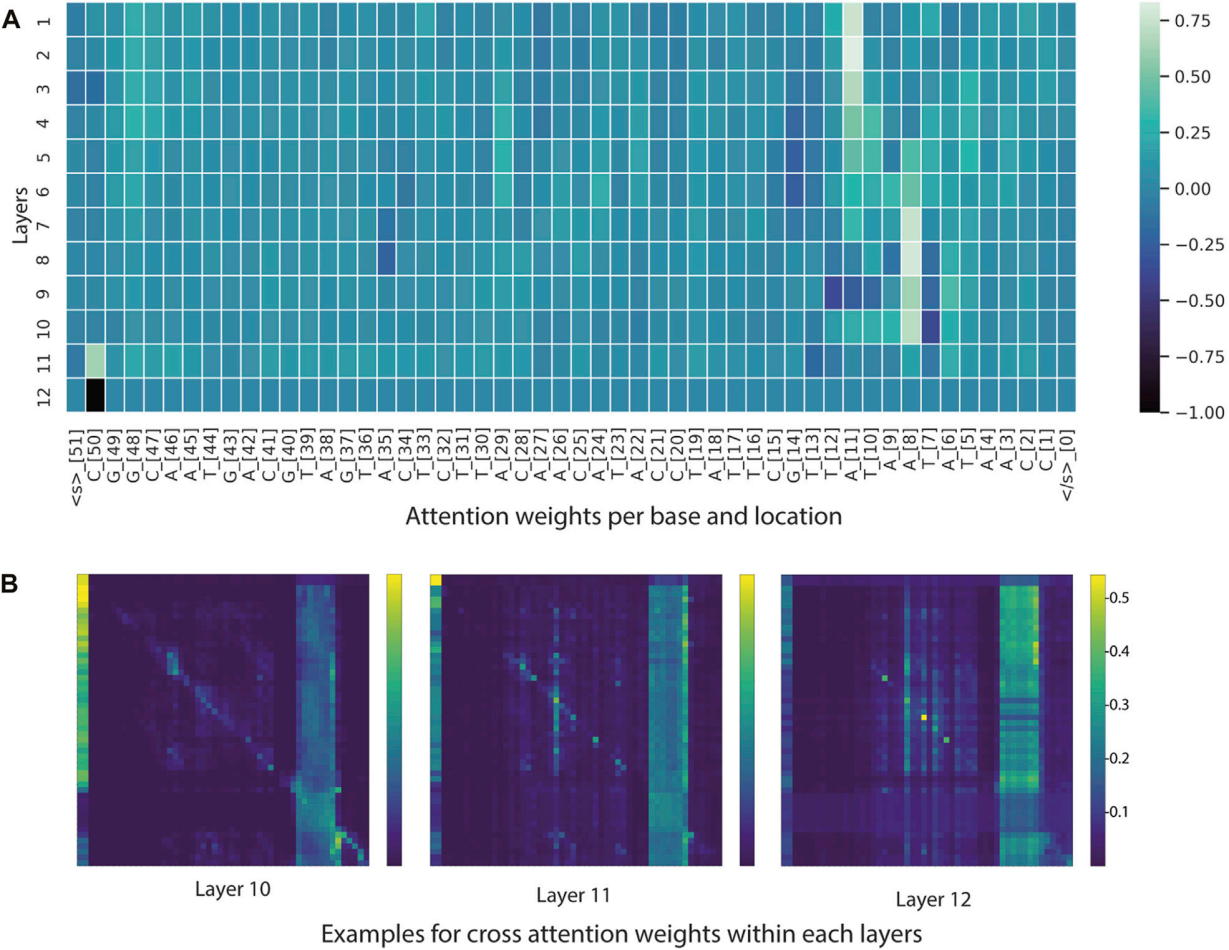
Visualization tools for model interpretability. **(A)** Heatmap based on attention scores of nucleotides (tokens) across 12 layers. **(B)** Heatmap illustrating cross-attention scores of nucleotides (tokens) in the last three layers. In the example heatmap, the self-attention modules focused on -10 element and some positions in the -35 element.

**FIGURE 7:**
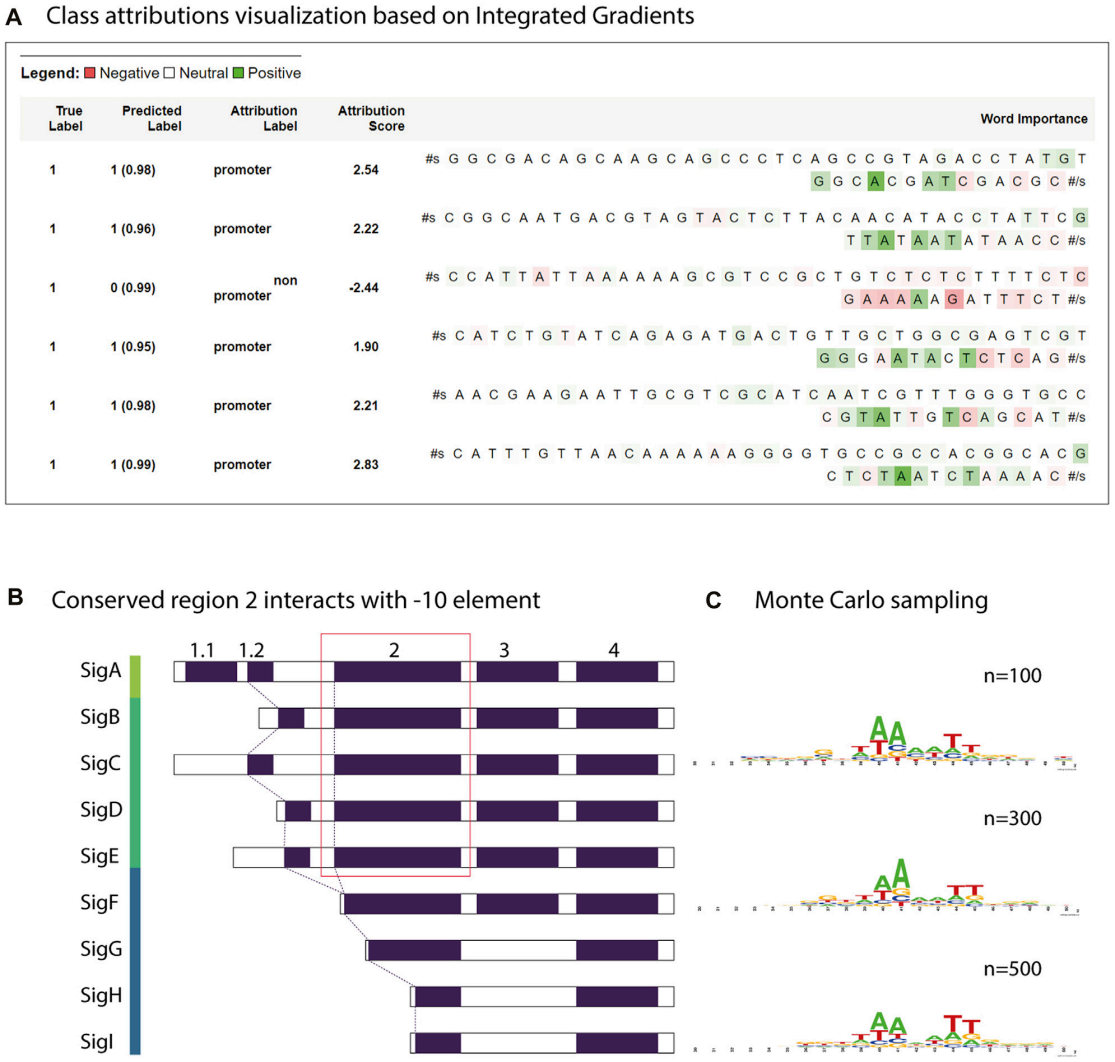
Motif analysis using attribution weights and reverse enrichment through Monte Carlo sampling. **(A)** Class attributions visualization of a few strong promoters in *Synechococcus elongatus* sp. UTEX 2973 and a non-promoter sequence. **(B)** Transcription factor groups in *Synechocystis sp.* PCC 6803. The relatively conserved region two in group 1 and group 2 retains a motif similar to the consensus -10 element TATAAT. **(C)** The motif learned by the trained model discovered by Monte Carlo sampling.

From the BERTviz model view and Captum, it appeared that a large number of self-attention modules focused on -10 element and occasionally on -35 element for sequences classified as promoters (Figure 6 and Figure 7). This is understandable, as the training dataset consists of all promoters from different sigma factor groups. In prokaryotes, the promoter regions are AT-rich and depend on the differences between their local structural properties and flanking sequences. The AT-rich -10 element plays a conserved role in DNA unwinding and facilitates transcription. Therefore, the constructed model could capture this local interaction context for promoter classification. Not all attention modules were utilized in the trained model; non-operational modes were observed in several layers and attention heads (cross-attention pointing to <s> and </s > tokens).

To estimate the closeness of the classifier to the real consensus of the -10 element, we defined a simple Monte Carlo generator using the constructed CyaPromBERT model as the discriminator. The pseudo-random generator generated fixed-length DNA sequences (50 nucleotides) until an expected number of sequences (500 sequences) passed the discriminator (cutoff value ≥0.99). Using this enrichment method, a recognition motif of the GnTAAAATT region was identified with a strong emphasis on thymine at the -11 and adenine at the -10 and -9 positions followed by two thymine bases at -6 and -5 (Figure 7C), which is similar to the consensus motif of the extended -10 element GnTATAAT of the extended -10 element previously reported in *E. coli* (Feklistov and Darst 2011). Further stretching of GGG was similar to that of the discriminator element in *E. coli*. Reversed enrichment using Monte Carlo sampling did not yield any motifs for non-promoter sequences. Promoters recognized by sigma factor groups have preferred motifs; however, crosstalk between groups does occur due to similarity of the transcriptional factors (Figure 7B). Group 1 (SigA), from the model cyanobacterium *Synechocystis* sp. PCC 6803 has consensus motifs similar to RpoD from *E. coli* (-35 element TTCACA and -10 element TATAAT), whereas the promoters recognized by sigma factor group 2 (SigB,C,D,E,F) have only a consensus motif of TATAAT for the -10 element. Group 3 (sigF,G,H,I) has dissimilar motifs of the -32 element TAGGC and -12 element GGTAA (Imamura and Asayama 2009). Therefore, the trained model CyaPromBERT potentially learned and gave better attention scores to nucleotide matching the enriched motif to distinguish promoter-like and non-promoter sequences.

### Cross-species validation through *Synechocystis* sp. PCC 6803 and *Synechocystis* sp. PCC 6714 datasets

As stated above, one of the main objectives of the current work was to use the limited dRNA-seq datasets of some model organisms that are closely related to the organisms of interest to construct curated models capable of high-performance inferencing for species with similar lineages by taking advantage of the learning transferability of deep-learning models. Therefore, we further validated the trained model using promoter and non-promoter datasets prepared from *Synechocystis* sp. PCC 6803 using TSSNote. They were from a different genus than *Synechococcus elongatus* sp. UTEX 2973. The trained model performed well on promoter prediction tasks using datasets consisting of 2840 sequences from *Synechocystis* sp. PCC 6803, with an AUROC score of 0.961 and F1 score of 0.91 (Table 4). A slight reduction in performance compared with that of *Synechococcus elongatus* sp. UTEX 2973 may be due to overfitting or differences in genomic preferences between the two species. Additionally, we trained similarly a promoter prediction model from *Synechocystis* sp. PCC 6803 and cross validated it with a closely related species *Synechocystis* sp. PCC 6714. The performance was similar but F1 scores of 0.89 were lower than those from *Synechocystis* sp. PCC 6803 (Table 5). However, it should be noted that the quality of datasets for *Synechocystis* sp. PCC 6714 was not high, leading to more noisy data. Regardless, the results still demonstrated the capability of maintaining good performance in cross-species promoter prediction from similar lineages.

**TABLE 4:** Cross validation the performance of CyaPromBERT trained on *Synechococcus elongatus* sp. UTEX 2973 for a distantly related species Synechocystis sp. PCC 6803.

Species	AUROC		Precision		F1 score		Support	
	Promoter	Non-promoter	Promoter	Non-promoter	Promoter	Non-promoter	Promoter	Non-promoter
*Synechococcus* sp. UTEX 2973	0.98	0.98	0.92	0.95	0.93	0.93	1001	1036
*Synechococcus* sp. PCC 6803	0.96	0.96	0.88	0.94	0.91	0.91	1407	1433

**TABLE 5:** Cross validation the performance of CyaPromBERT trained on *Synechocystis* sp. PCC 6803 for a closely related species *Synechocystis* sp. PCC 6714.

Species	AUROC		Precision		F1 score		Support	
	Promoter	Non-promoter	Promoter	Non-promoter	Promoter	Non-promoter	Promoter	Non-promoter
*Synechococcus* sp. PCC 6803	0.97	0.97	0.91	0.92	0.91	0.92	364	378
*Synechococcus* sp. PCC 6714	0.96	0.96	0.91	0.88	0.89	0.89	330	330

### The limitations of the pipeline and the trained model

Despite the fast construction and relatively high performance, a few limitations were present in the current work. First, for TSSNote, the quality and accuracy of promoter extraction depend on the quality of raw dRNAseq datasets and their experimental designs. The quality and performance of the trained model also depend on the quality of the inputs; therefore, selecting suitable parameters and preparing good datasets are the most important part of this pipeline. We tested the pipeline on datasets of the model acetogen *Eubacterium limosum* (Song et al., 2018). The pipeline produced a model with F1 scores of 0.88 and AUROC scores of 0.89. However, when we tested the pipeline on more dated datasets of other species, the trained models did not perform well. Second, despite the high performance of the test datasets and cross-validation, the trained model still suffers from false positives in the regional scanning mode. Thus, the results should be interpreted as the most potential locations, and further analyses for decision-making should be conducted. There are several possible explanations for this finding. To capture most promoters of the genera *Synechocystis* and *Synechococcus* through the learned pattern, the model focused solely on the interrelationship and composition of nucleotides in the -10 element. Therefore, the model may be confused with AT-rich promoter-like sequences. Another explanation is that transcription is a complex biological process, which is influenced by multiple factors, such as protein–DNA interactions and protein–protein interactions (DNA-binding proteins, transcription factors, enhancers, competition of sigma factors for the holoenzyme RNA polymerase), and the topographical state of the genome (chromosome folding states). The tertiary structures of chromosomes can greatly influence functional DNA-related processes, such as transcription and DNA replication (Dorman, 2019; Szabo et al., 2019). Such interactions cannot be fully captured with sequential information, which is another limitation of the current work. Regardless, the transformer architecture is a powerful building block for the construction of multimodal models; therefore, future incorporation of additional data reflecting cis/trans interactions and/or other neural networks may improve the accuracy and reduce false positives to make the model more deterministic. The pipeline and model in the current work may be used for constructing a fast and accessible promoter prediction and screening tool using a deep-learning approach, which can help reduce the time needed for downstream analyses.

## Conclusion

With the rapid evolution and continuous development of next-generation sequencing techniques, an unprecedented vast amount of high-quality biological data has become increasingly accessible to researchers. This ever-expanding source of genomic data is a valuable, yet underexplored, reservoir of knowledge that can provide valuable insights into the mystery of life. Recently, methodological and computational advancements have enabled systematic and high-throughput approaches to elucidate the biological meanings of DNA sequences, in addition to traditional knowledge-based analysis. The traditional method for promoter identification involves dRNA-seq or 5′-CAGE experiments. However, despite the growing number of high-quality RNA-seq datasets, dRNA-seq experiments are still limited and expensive. In the current study, we applied and compared the performance of various SOTA transformer-based models for promoter prediction of *Synechococcus elongatus* sp. UTEX 2973 *and Synechocystis* sp. PCC 6803. The model achieved an AUROC score of 97% and an F1 score of 92% in the validation dataset of the promoters extracted from *Synechococcus elongatus* sp. UTEX 2973 and had an AUROC score of 96% and F1 score of 91% when cross-validated using 7000 promoters from *Synechocystis* sp. PCC 6803. This finding illustrated that core promoter features are conserved in related species, and the dRNA-seq dataset of one model organism is sufficient to construct a curated promoter prediction model.

Precise promoter prediction is essential to understand the regulatory mechanisms of genes and operons. A key advantage of this study is that it can rapidly identify potential promoter sequences and regions from genomic data with high precision. The model is integrated with the visualization libraries BERTviz and Captum to visualize cross-attention weights, allowing closer observation of base-pair interactions. To increase accessibility to other researchers, both the models and pipeline were ported to the cloud-computing service Google Colab. The pipeline developed (TSSNote and PromBERT) in this study can be applied to other species and lineages to develop fast promoter prediction tools. As transformer architecture has become increasingly popular for multimodal learning, the implementation and analysis of BERT behavior in the context of genomics is another case study for developing more robust implementations of transformers for biological application.

## Data Availability

The datasets presented in this study can be found in online repositories. The names of the repository/repositories and accession number(s) can be found in the article/supplementary material.
